# Mapping Evidence on the Regulations Affecting the Accessibility, Availability, and Management of Snake Antivenom Globally: A Scoping Review

**DOI:** 10.3390/tropicalmed10080228

**Published:** 2025-08-14

**Authors:** Ramsha Majeed, Janette Bester, Kabelo Kgarosi, Morné Strydom

**Affiliations:** 1 Department of Pharmacology, School of Medicine, Faculty of Health Sciences, University of Pretoria, Pretoria 0002, South Africa; morne.strydom@up.ac.za; 2 Department of Physiology, School of Medicine, Faculty of Health Sciences, University of Pretoria, Pretoria 0002, South Africa; janette.bester@up.ac.za; 3 Department of Library Services, Sefako Makgatho Health Sciences, Pretoria 0204, South Africa; kabelo.kgarosi@smu.ac.za

**Keywords:** snakebite envenoming, antivenom, regulations, availability, accessibility

## Abstract

The World Health Organization (WHO) declared snakebite envenoming (SBE) as a neglected tropical disease in 2017. Antivenom is the gold standard of treatment, but many healthcare barriers exist, and hence, affected populations are often unable to access it. The challenge is further perpetuated by the lack of attention from national health authorities, poor regulatory systems and policies, and mismanagement of antivenom. This study aims to map the evidence regarding snake antivenom regulations globally and identify gaps in the literature to inform future research and policy. This review was conducted using the original Arksey and O’Malley framework by three independent reviewers, and the results were reported using the Preferred Reporting Items for Systematic reviews and Meta-Analysis Extension for Scoping Reviews (PRISMA-ScR). A search strategy was developed with assistance from a librarian, and six databases were searched: PubMed, SCOPUS, ProQuest Central, Africa Wide Web, Academic Search Output, and Web of Science. Screening was conducted independently by the reviewers, using Rayyan, and conflicts were resolved with discussions. A total of 84 articles were included for data extraction. The major themes that emerged from the included studies were regarding antivenom availability, accessibility, manufacturing, and regulations. The study revealed massive gaps in terms of policies governing antivenom management, especially in Asia and Africa. The literature does not offer sufficient evidence on management guidelines for antivenom in the endemic regions, despite identifying the challenges in supply. However, significant information from Latin America revealed self-sufficient production, involvement of national health bodies in establishing efficient regulations, effective distribution nationally and regionally, and technology sharing to reduce SBE-related mortality.

## 1. Introduction

Snakebite envenoming (SBE) affects many people worldwide. The high levels of mortality have turned it into a significant public health challenge [1]. Over 2.7 million people are estimated to suffer the consequences of SBE annually, with up to 138,000 fatalities and 400,000 victims left with disabilities [2,3]. In 2017, the World Health Organization (WHO) created a comprehensive strategy to combat SBE, with the aim to reduce its impact by ensuring the availability of and access to antivenoms globally by 2030 [2,3].

Snake venom is composed of a complicated mixture of varied toxins. The damage caused to victims could be either local or systemic, while many people are left with permanent disability [4]. Clinical symptoms differ and are based on snake species, venom composition, and bite size. Common signs include myonecrosis, compromised vascularity, inflammation, pain, immobility in limbs, acute kidney injury, and neuromuscular paralysis of muscles in the respiratory system, neck, and limbs. This can progress to shock, organ failure, and eventually death [5]. Backed by 120 years of research, antivenom is considered the mainstay therapy for SBE [6]. It is prepared by immunizing animals such as horses and sheep [7]; the polyclonal immunoglobulins produced in the animals’ plasma are collected and purified to obtain F(ab’)2 and Fab fragments [8]. Antivenoms are also produced from whole IgG molecules [9]. The discovery of antivenom is attributed to the work of Albert Calmette, who laid down the foundation of antivenom production from animal immunization. His work was further refined to commercialize antivenom [6,10].

Snakes largely populate warmer tropics and subtropics; hence, the burden of snakebites is concentrated in the rural areas of Asia, sub-Saharan Africa, South America, and Oceania [11]. The disproportionate impact of SBE on poor communities is a compounded result of geographical and socio-economic factors [11]. Risk factors include occupations such as agriculture, farming, fishing, and herding, which increase exposure to snakes. Additionally, those living in less developed areas with proximity to snake habitats are likely to be affected [12].

Despite the existence of a standard and effective treatment, SBE remains a significant challenge due to many reasons. Data paucity exists, and despite the large-scale incidence globally, epidemiological information remains unreliable or unavailable [12]. This data is crucial in establishing SBE management guidelines, planning the allocation of healthcare resources, providing essential training to healthcare professionals, and having policies in place to improve antivenom availability and access [12]. In rural areas, which are widely affected, there is a low literacy rate and a wide education gap; hence, victims are often unaware of appropriate treatment for snakebites and the usage and availability of antivenom and prevention measures [13]. The SBE problem is compounded by health–economic factors. Due to the lack of long-term investment in health systems and market failure, there are shortages of high-quality and affordable antivenom [14]. The lack of health investment and poor health infrastructure in SBE endemic regions have lowered antivenom production and distribution capacity [15].

Victims are unable to receive timely treatment due to the absence of transport facilities, long and uncrossable distances to hospitals and clinics, and logistics that are not in their favor [13]. As a result, many victims choose to seek treatment from traditional healers and rely on complementary and alternative medicines (CAMs), as these provide easy and cheap access to treatment [13]. The cost of antivenom is an added factor to this behavior, as the expense is unaffordable for many victims, and they are often left in debt or lose assets [16].

The biggest challenge is the imbalance between antivenom demand and supply. There is a chronic shortage of this life-saving medicine globally, owing to gaps in production, regulations, and accessibility [17]. The WHO attributes the poor management of SBE to inadequate regulatory frameworks, which provide guidelines extending from antivenom production to the end stage, where it is administered to patients [18]. Despite it being recognized as a neglected tropical disease, SBE remains overlooked by national health authorities, and no significant actions have been taken to solve the antivenom supply crisis [14]. The lack of funding in healthcare, socio-economic factors, and neglect by policymakers have only allowed the burden to grow, especially in communities that are unable to access antivenom [14]. There is an urgent need for solutions geared towards solving the antivenom supply challenge. It is important to recognize the role of regulatory bodies and systems that govern antivenom availability and accessibility to victims, especially in regions heavily burdened by SBE.

A scoping review identifies gaps in the literature, maps out key concepts and theories, and provides implications for future research and policy. For a qualitative study to explore complex and heterogeneous topics, this is an excellent research tool [19]. This scoping review aims to determine if sufficient evidence exists in the literature, based on the inclusion criteria, and to use the results to identify gaps in the literature regarding the regulation of antivenom globally to guide future research and policymaking.

## 2. Materials and Methods

### 2.1. Study Design

This scoping review is a component of a larger study titled: Identifying and exploring the regulatory framework gaps affecting accessibility, availability, and management of snake antivenom in South Africa. This review was registered at the Open Science Framework (OSF) and can be found at the following web address: https://osf.io/54zja (accessed on 1 May 2025). The scoping review protocol was published in BMJ Open [20].

The review was performed using Arksey and O’Malley’s framework developed in 2005 [21], with modifications and adjustments from the Joanna Briggs Institute (JBI) [22]. The review was conducted by three independent reviewers, and the screening results were reported using PRISMA-ScR [23]. The population, concept, and context (PPC) framework was employed to establish the eligibility criteria; see Table 1.

### 2.2. Identifying the Research Question

The research question led to the structure of the study and defined the inclusion and exclusion criteria. For this scoping review, the research question is: “What are the regulatory aspects affecting the availability and access of registered snake antivenom products globally?”

### 2.3. Identifying Relevant Studies

The PCC criteria helped in the development of the eligibility criteria.

Studies on regulatory aspects for registered snake antivenom were included globally, with no limitations on the location of publishing. Studies between 2009 and 2023 were included, because the WHO first acknowledged SBE as a neglected tropical disease in 2009, removing it in 2013 and then re-adding it to the list in 2017. Only studies published in English were considered due to a lack of translation resources.

The search strategy was built on PubMed with assistance from a health sciences librarian, followed by a pilot search on SCOPUS. Controlled vocabulary, keyword, and medical subject heading (MeSH) terms were used to create a comprehensive search. Six databases were used to conduct the search: PubMed, SCOPUS, ProQuest Central, Africa Wide Web (via EBSCO), Academic Search Output (via EBSCO), and Web of Science (Core Collection). Duplicates were removed using EndNote, and a total of 4075 articles were obtained. The comprehensive search strategies can be found in Supplementary Materials.

### 2.4. Study Selection

There were three reviewers who screened the studies independently, using Rayyan. A screening brief was developed to ensure consistency with the PCC framework. The first round of screening included titles and abstracts, while round two included full-text articles. Conflicts were resolved with discussions between the rounds.

### 2.5. Data Extraction and Charting

A Google form was developed to extract data. This was an iterative process, and the form was updated based on information available. The process was piloted for the first 10 articles before implementation. Data extraction was conducted by the principal investigator, while the other reviewers assessed the quality of information. After extraction, the output was obtained in the form of a Microsoft Excel document.

### 2.6. Collating, Summarizing, and Reporting Results

Data analysis was primarily performed in Microsoft Excel. Descriptive results were obtained; these included year of publication, type of publication, country of publication and income level, and concepts included. These results were presented visually using graphs and a map. Content analysis was conducted to provide evidence on major concepts: antivenom access and availability, manufacturing, and regulations.

## 3. Results

### 3.1. Screening Results

**Figure 1:**
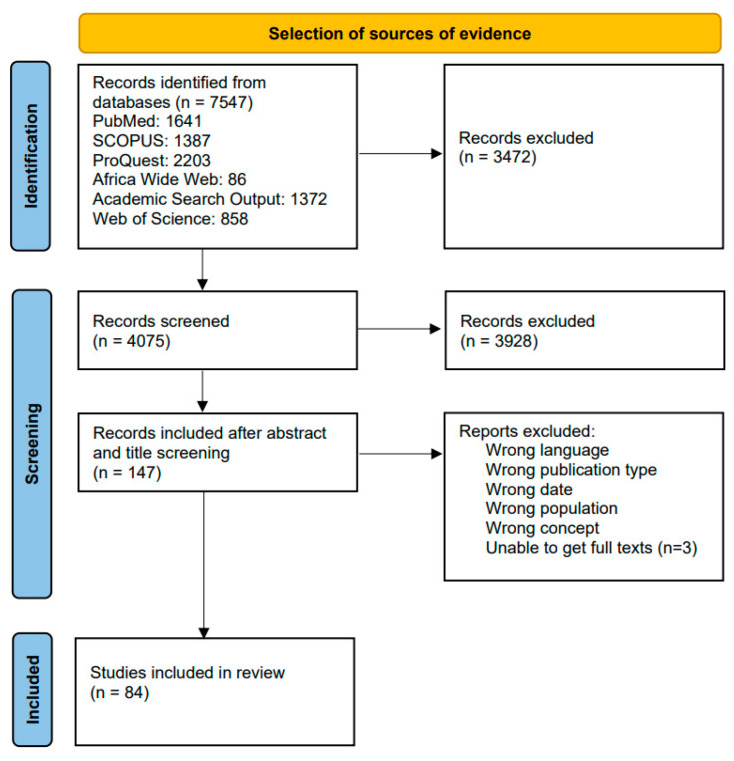
A flow diagram showing selection of sources for inclusion.

The search results had a total of 7547 records; after duplicate removal from EndNote, 4075 articles remained. After the first round of screening, 3928 articles were excluded. Full-text screening was performed for 147 articles, and, finally, 84 were included for data extraction. The selection of sources can be seen in Figure 1. During the screening process, conflicts between reviewers were resolved through consensus.

### 3.2. Descriptive Characteristics

#### 3.2.1. Year of Publication

**Figure 2:**
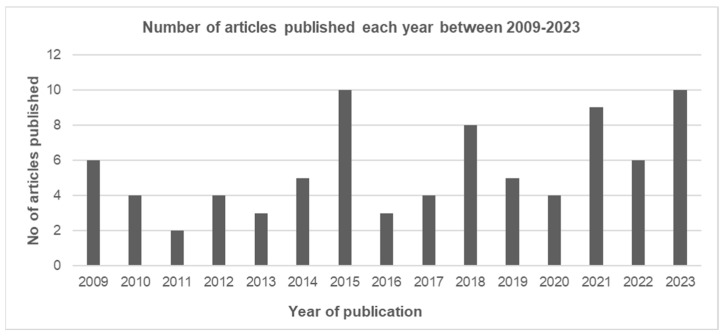
Number of included articles published per year between 2009 and 2023.

The search for this study was limited between 2009 and 2023. The year of publication for records included showed a varied trend. Both 2015 and 2023 showed the highest number of articles published: 10. A significant number of publications were also published in the years 2018, 2021, and 2022. The publications per year can be seen in Figure 2. Only two articles were published regarding snake antivenom in 2011. The year of publication was unavailable for one article.

#### 3.2.2. Type of Articles

**Figure 3:**
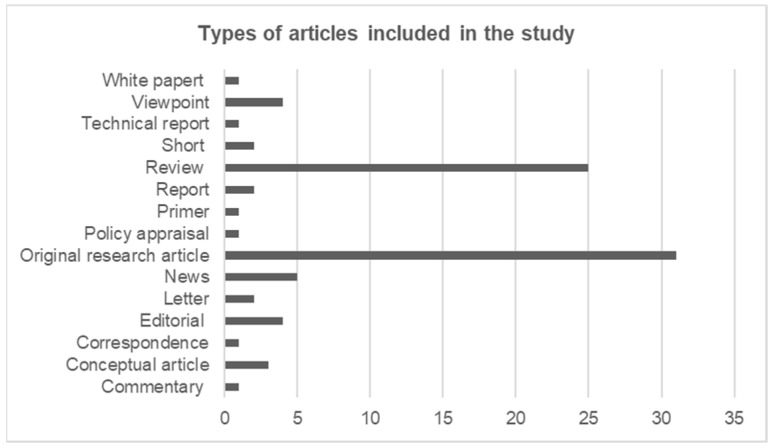
Number of publications included in terms of categories.

Out of 84 publications, 31 were original research articles, and 24 were review articles, the two making up 67% of the inclusions. The rest of the publications varied categorically, which included editorials, five news articles, and four viewpoints. The type of publications included in the study can be seen in Figure 3.

#### 3.2.3. Country of Publication and Income Level

**Figure 4:**
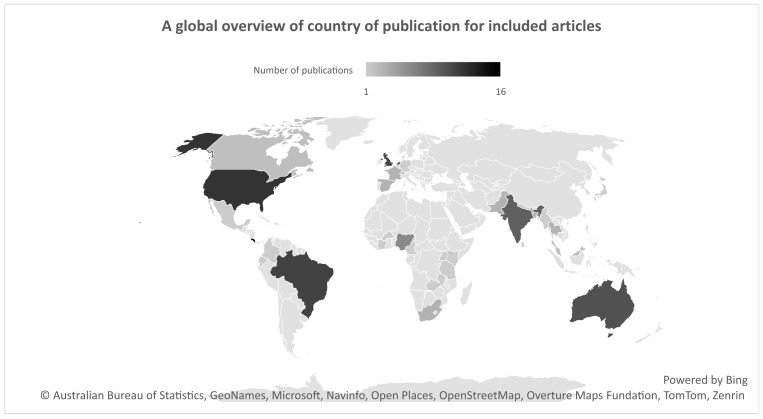
Number of publications from each country regarding snake antivenom regulations.

The country of publication was obtained through author affiliations, and, hence, some publications had multiple authors and affiliations thereof. Figure 4 shows an unequal distribution of studies globally. A high concentration of publications originated from high-income and developed countries with a low risk of snakebites: the USA leading with 12, followed by the UK with 11. Other countries with a high number of publications and a high snakebite incidence were Australia with 10 records and Brazil with 11. Costa Rica had the highest number of published studies (16) compared to the other countries. Regionally, a small number of publications resulted from Europe, Asia, and Africa. Out of the 84 records, 12 publications did not have a country of publication.

The income levels of the countries were dictated by categories from the official World Bank database. Fifty-five publications were attributed to high-income countries, while on the other extreme, only three articles originated from low-income countries. The distribution between upper- and lower-middle-income countries is more uniform, with 38 and 34, respectively.

#### 3.2.4. Concepts Included

**Figure 5:**
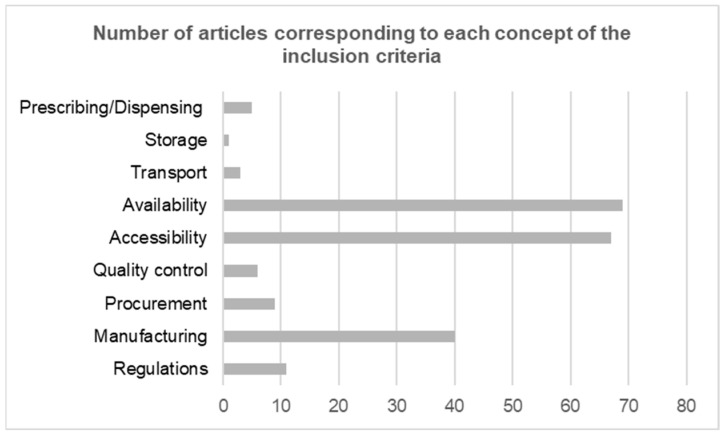
Number of articles presenting evidence on each concept from the PCC criteria.

The concepts listed in Figure 5 were part of the inclusion criteria, according to the PCC framework. The most inclusive concepts in the articles selected were availability (69) and accessibility (67). This was followed by 40 articles presenting evidence on manufacturing, being the third largest concept. Other concepts were not widely discussed, with procurement, quality control, and transport having less than 10 articles. Only one article included information regarding the storage of antivenoms.

### 3.3. Findings of the Study

The summary of key findings of the study can be found in Table 2.

#### 3.3.1. Regulatory Frameworks

Several articles presented comprehensive evidence on the national health systems of Brazil, which heavily relies on gathering data on SBEs to combat the problem. The Ministry of Health has developed systems to determine antivenom need and production thereof, ensuring distribution to all health units. Additionally, the government has placed programs to provide free-of-cost antivenom to patients. The health ministry, together with the four major manufacturing labs, coordinates antivenom production at a national level [24]. The acquisition is centralized by the ministry; the antivenoms are first received by the National Centre for Distribution and Storage of Immunobiologicals (CENADI). Quality control is performed by the National Institute for Quality Control in Health (INCQS) [25]. Distribution to the localities and municipalities is decentralized. Serum distribution is conducted by the health ministry; meanwhile, the secretaries of health for each state determine the areas that receive antivenom. The Instituto Vital Brazil’s official web page has the list of hospitals that receive antivenom from the distribution network of the government; however, the specificity of the antivenom is unavailable [26,27].

Sources of evidence also describe regulatory systems in South America, where antivenom is mainly manufactured by Costa Rica, Mexico, and Argentina. These countries export antivenom across the region, facilitated by health ministries and other public health institutes, which have developed purchasing schemes to procure these essential medicines regularly. In Costa Rica itself, distribution to health facilities is carried out at specific levels of specialization (hospitals, clinics, primary care centers) [28].

Countries in this region have similar regulatory systems, whereby the Ministry of Health plays the defining role. Argentina’s Ministry of Health receives locally produced antivenom, which is then provided to the Zoonoses section of the ministry (central level). The central level further allocates the antivenoms to jurisdictional referents (intermediate level) and eventually to the provincial healthcare facilities (local level) [26]. Paraguay (relying on imported antivenom) has a decentralized acquisition process at a local level, but no quality control is performed on the products [26]. Bolivia does not have a national program to coordinate SBE management or facilitate antivenom distribution [26]. Ecuador also relies on its health ministry to acquire and distribute antivenoms, but the National Control for Biologics performs quality assurance on imported products. After inspection, they are stored by the National Bank of Vaccines, and distribution is performed via regional sub-secretaries. Costs of antivenom are determined by the National Council for the Fixation and Prices of Medicines (a subsidiary of the Ministry of Public Health) [26].

In Colombia, the Ministry of Health and Social Protection plays a crucial role in determining antivenom shortage and needs. The major regulatory organization is the National Institute for the Surveillance of Medicines and Food (INVIMA), which authorizes the registration of antivenoms and performs regular quality assurance. Circulation is coordinated by health insurance companies and the Ministry of Social Protection [26]. Panama imports antivenoms, based on acquisition protocols by the national health systems. Upon purchase, they are stored, and quality assurance is performed. The final product distribution is conducted to provide antivenom locally across the country [26]. Nicaragua’s Ministry of Health imports antivenom from Costa Rica, and it is included in the Basic List of Medicines. Distribution is provided to three health units: national reference hospitals and regional hospitals, health centers with beds, and the primary healthcare units at local levels that have physicians [26]. Antivenoms are included in the official list of essential medicines by Costa Rica. After local production, the quality control laboratory certifies the product. Antivenom is distributed to three levels: national hospitals, major clinics and peripheral hospitals, and primary healthcare units [26].

Not much evidence was found for the Asian continent. In India, the government has laid out the protocols required to manufacture antivenom. Snakes are protected under the Wildlife Protection Act; hence, venom extraction cannot be conducted without approval from state wildlife authorities [29]. In Bangladesh, the government procures and imports antivenom from India and distributes it free of cost in public hospitals [30]. Thailand has a National Antidote Programme, responsible for stocking and distributing national and subnational antidotes (including antivenoms). The stocking is based on a web-based system, which determines the demand and supply. The National Health Security Office finances and manages procurement [31].

Evidence on the African continent was also scarce. The sole antivenom manufacturer in South Africa has no commercial export policy for public scrutiny, according to the included literature [32]. Burkina Faso made SBE notifications operational in 2010. Regulations are in place to ensure antivenom is only sold by licensed wholesalers [33]. In Rwanda, the Rwanda Medical Supply is a centralized store that procures medicines and distributes them to pharmacies and government health facilities. Non-government facilities procure medicines from private wholesalers, especially if the central store is unable to meet demand [34].

In the USA, the Food and Drug Administration (FDA) is responsible for approvals and distribution of essential medicines. A few articles cited the approvals made by the FDA to use expired coral snake antivenom, considering the limited supply and no alternatives present [35,36]. In the Netherlands, antivenoms are not registered. Instead, the Netherlands Health Care Inspectorate gives approval to the National Institute for Public Health and the Environment to import, store, and distribute antivenoms. These activities are guided by the Good Distribution Practice (GDP). They are only distributed to hospitals, and physicians are required to sign a declaration of awareness, stating they are aware that antivenom is an unregistered product and has potential risks associated with its use [37].

#### 3.3.2. Manufacturing and Procurement

The studies identified several major producers of antivenom globally, as well as countries that do not have local manufacturing laboratories and rely on imports. The manufacturers are heterogeneous with regards to technology, training, the application of good manufacturing practices (GMPs), production volumes, quality control, and distribution ranges [38]. According to the WHO, there are 46 laboratories worldwide that produce animal-derived antivenoms, the majority located in Asia and the Americas [39].

Amongst the high-income countries, the USA, the UK, France, Australia, and Japan manufacture antivenom. In Latin America, Costa Rica, Brazil, Argentina, Colombia, and Mexico have functional antivenom-producing facilities. The largest producers in the region are Instituto Clodomiro Picado in Costa Rica and Instituto Butantan in Brazil, and they also provide the product to Panama, Nicaragua, Honduras, El Salvador, Guatemala, Belize, Trinidad Tobago, Island of Saint Lucia, and Ecuador. Other countries with antivenom-producing laboratories include Peru, Venezuela, and Bolivia [28,40,41,42]

In south Asia, India is the major antivenom producer, and it provides the product to the neighboring countries Bangladesh, Pakistan, Burma, and Nepal [43]. Meanwhile, in Southeast Asia, Thailand, Indonesia, and the Philippines manufacture antivenom. Lao PDR relies on Thai imports [31,44,45]. South Africa (South African Vaccine Producers) and Egypt (Vacsera) are the sole antivenom producers on the African continent, while the rest of the region including countries depend on imports [46,47]. Papua New Guinea receives its antivenom supply from Australia [48].

#### 3.3.3. Availability and Access of Antivenom

The included studies examined the imbalance between antivenom demand and supply. Supply is limited, especially in LMIC, due to disruptions and an overall lack of production. Meanwhile, demand is not fully reported and is low due to a smaller number of patients in healthcare settings. The reviewed studies highlight manufacturing gaps that exist due to major antivenom suppliers ceasing their production in recent years. These include Sanofi-Pasteur, Wyeth, Syntex, and Behringwerke [49,50,51]. The decision to halt antivenom production is attributed to economic constraints, market failure, lack of commercial incentives for manufacturers, and low profits. Funding provided to antivenom production is not cognizant of the high manufacturing costs. These expenses come from animal husbandry, manufacturing processes, stocking, and quality control systems. As a result, some antivenoms (Sanofi-Pasteur’s Fav Afrique) were too expensive for the market saturated with cheaper alternatives, leading to low demand and hence insufficient production [42,52,53,54,55,56].

In the face of shortage, expired antivenoms are reported as an effective, safe, and valuable resource. One study provided evidence of expired products being used in ASEAN countries. Another study reported that the FDA extended expiration dates for coral antivenoms to combat limited supply, after manufacturing ceased. Antivenom (11 years past expiry) was also supplied by Liverpool School of Tropical Medicine, by maintaining a cold chain [31,36,45,57]. Five articles linked antivenom shortages to COVID-19, citing problems such as production halts, decreased budgets, supply chain disruptions, and increased import times [58,59,60,61].

Access is hindered by the failure to maintain a cold chain, as stability and refrigeration are difficult. The product has a limited shelf life; hence, it expires quicker, and there is a lack of profitability [62,63,64]. There is poor distribution to endemic areas. Some countries lack central supply systems, while in others, geographic inaccessibility is a challenge. Due to poor health infrastructure and overall economic constraints, lack of transport and healthcare facilities equipped with adequate stores further delay access. Even if antivenom is purchased from other countries, it does not guarantee deployment [42,51,52,59,65,66]. Six studies reported exemplary distribution practices by Latin American countries, especially Brazil and Costa Rica [24,27,28,53,58,67].

Several articles explored the challenge of antivenom costs, which limit accessibility to patients [68,69]. High costs are due to preclinical studies, maintaining snakes, production expense, following accurate standards when manufacturing, and assuring quality. Additionally, patients may require multiple vials for one incident of SBE, adding to the expense of healthcare. Often, the victims and their families are unable to meet the threshold of affordability [14,15,34,42,59]. Two studies reported the availability of free-of-cost antivenom in Brazil [24,70]. In Burkina Faso, the expense problem was solved by lowering the prices of antivenom by public wholesalers with government assistance [33]. The universal health coverage in Thailand has also promoted access [71]. In India and Pakistan, cheap antivenom is available, but overall treatment is expensive [29,72].

## 4. Discussion

The purpose of this scoping review was to map the literature for published guidelines that control victims’ access to and the availability of antivenom worldwide. The findings revealed a concentration of studies discussing antivenom access and availability, manufacturing, and regulations, with limited attention to other concepts. Geographically, most research originated from the USA, the UK, Australia, and Latin America, while scarce publications were found from Africa and Asia.

The findings of this study are consistent with those of Matlani [17], who emphasized that the major hurdles against antivenom availability and access are owed to the complex landscape of SBE management. These challenges include lack of production, weak regulatory frameworks, poor-quality products circulating in the markets, underreporting, and poor health infrastructures. Neumann et al. [64] adds that the lack of improvement for SBE outcomes is largely owed to data paucity and lack of antivenom availability. There is scarce data on antivenom distribution and use in sub-Saharan Africa. The product is unavailable, especially in rural areas with the higher incidences [64].

This study presented high costs as a major obstacle for antivenom access. While the main production strategy for antivenom has remained the same since it was first established, the technological improvements over time have significantly improved envenoming therapy and have led to higher-quality products [73]. Bureaucracy, patents, and litigation also adds to the cost. While it remains the single most effective treatment for SBE, the costly treatment has caused medical debt due to the extraordinary expenses. It should be noted that this includes not only the medicine itself, but also transportation, hospital stay, healthcare visitation, and possible follow-up treatments [16]. Healthcare costs are a cause of concern for rural populations, especially in LMICs, and the affordability threshold is directly linked to the mortality due to SBE. The high treatment costs also impact the decision-making process and treatment-seeking behavior of victims. And even those who do seek the appropriate treatment are often left with long-term financial consequences. For example, lack of insurance or poor affordability leads to many selling valuable assets to meet treatment demands [74].

There was a notable trend of studies highlighting usage of expired antivenom, in light of the supply crisis. Soopairin et al. [75] notes that the limited access to antivenom is a global challenge, and expired antivenom is used to fill the gaps, however, with the risk of compromising safety and quality. The shelf life of antivenoms is between 2 and 5 years, and normally, they expire before use. The primary cause of wasting extremely valuable antivenom is due to the challenges of managing national and regional antivenom inventories, which are being actively addressed by several stakeholders. These challenges include a lack of knowledge and research regarding national requirements, inadequate infrastructure, and financial inaccessibility [76]. Recent studies have tapped into determining the feasibility and clinical efficacy of expired antivenom as a valuable resource to combat the shortage. For instance, although its manufacturer Wyeth terminated the use of expired North American coral snake antivenom in 2006, the US Food and Drug Administration (FDA) authorized its use [76]. Several studies have confirmed the stability and efficacy of lyophilized antivenom for up to 20 years. Lao PDR’s case of using expired antivenom is highly documented [76]. For example, a study demonstrated that Japanese antivenom against Yamakagashi (*Rhabdophis tigrinus*) was physiochemically stable even 20 years past expiry. The United States produces lyophilized antivenom against the North American coral snake (*Micrurus fulvius*) to prevent rare incidents of coral snake envenomation. The product’s original 5-year shelf life has since been extended by 12 years. Additionally, the lyophilized Thai snake antivenom made at QSMI in Bangkok has a five-year shelf life. Preclinical laboratory testing, however, verified continued efficacy seven years from the date of manufacturing [45].

While the study assessing 20 antivenom products by Soopairin et al. revealed evidence of preclinical efficacy, there is still a notable lack of data supporting the safety and effectiveness of expired products [77]. The stability profiles of antivenoms vary depending on the formulation; hence, the usage of expired antivenom cannot be generalized [77]. The antivenom manufacturers need to undertake long-term studies of product stability, safety, and maximization of shelf lives. The national regulatory authorities can use this information to conduct benefit–risk analysis on a case-by-case basis and make informed decisions regarding off-label use of expired antivenoms in cases of unavailability [77]. Additionally, usage of expired antivenom should remain an exception, as over reliance could negatively impact efforts to build stronger supply chains and combat stockouts [77].

The lack of studies addressing regulatory gaps in the endemic regions of Asia and Africa is a cause of concern and reflects a significant knowledge gap. Despite the disproportionately high burden of snakebites in Southeast Asia, with envenomation between 78,000 and 470,000 annually, management of antivenom and access to patients remains a challenge [31]. This is attributed to a lack of sound pharmaceutical logistic systems to support distribution, the high costs of antivenom in some settings, insufficient production, insufficient information systems to report SBE, and the lack of sufficient supply to some countries such as Lao PDR [31,78]. Costs are high, largely due to imported antivenom [31]. Beyond the issues of availability and access due to insufficient manufacturing capacities in ASEAN countries, Patikorn et al. [78] emphasizes the need for strong health management systems, health infrastructure, the involvement of communities to seek appropriate treatment, rational use of antivenom, and rigorous regulatory systems.

The biggest challenge of the African continent is the security of the supply, with only the SAVP (South Africa) being the major producer and regional distributor of antivenom [15]. The rest is supplied by producers in Asia, Europe, and Latin America, largely on a business commercial model. As a result, costs are high, and some countries are only able to afford small amounts of antivenom that fail to fulfil the demand [15]. Imported products are not always assessed for quality. For instance, significant mortality was reported due to antivenoms used in Chad, Central Africa Republic (CAR), and Ghana, after Fav-Afrique stockouts [15].

Although most African nations have regulatory bodies, their ability to control, assess, and prove the efficacy of the goods and the safety is still not adequate. The capacity of regulation and quality assurance is a key challenge in the development of biomedical products, as manufacturers need proper regulatory frameworks for product approval and appraisal. This has resulted in a flood of sub-standard antivenom in the market, leaving little space for quality products and resulting in misconceptions and mismanagement [79]. The sub-standard products in the market have had a lasting impact, diminishing the confidence of health forces in antivenom treatment. Upon halting production of Fav-Afrique, Sanofi quoted limited profits and market failure in Africa as the main reasons behind the cessation [80].

While many toxin families represent the same clinical profiles, there still exist inter- and intra-specific variations in the compositions of the snake venoms; hence, venom is unique to each snake species [81]. In the absence of local manufacturers, many countries turn to imports, despite the geographical distribution of snake species being specific to each country. Polyvalent antivenoms are considered a desirable option; however, in the case of reduced antibody specificity, larger doses are required, and hence, costs increase [81]. In Asia, the region with the highest burden of SBE, the major antivenom exporters are India and Thailand [81,82]. It should be noted that Indian polyvalent antivenom has limited clinical efficacy against identical species in various regions of the country [82]. Meanwhile, on the African continent, Algeria, Tunisia, Egypt, and South Africa are the only exporters listed in the WHO database, leaving the region to rely heavily on imports, which adds to unstable supply chains and high costs. Additionally, there is limited evidence on the approval, efficacy, and quality control of products imported in many African countries [82].

The success of imported antivenoms is limited due to the biodiversity of snake species, venomics, the clinical ineffectiveness of products, and the lack of procurement and distribution policies. The imported antivenoms have reduced coverage and may not target all snake species, irrespective of medical importance [82]. The reliance on imported antivenoms adds to a significant healthcare cost, especially when countries expend their foreign currency on antivenom, making the final product unaffordable for patients [15].

Significant studies on Latin America reflect concentrated regional efforts to reduce the SBE burden. Important progress has been made to understand SBE and the role of antivenom in eradicating the problem. The healthcare sector in the region has been transformative in the previous decade, with an exemplary increase in health spending and coverage, and the launch of successful public health initiatives [83]. The work of Vital-Brazil at the Instituto Butantan served as a pioneer of the long-standing antivenom production in South American countries. The region has achieved a great degree of self-sufficiency in terms of manufacturing national antivenom and filling the gaps with technology and knowledge transfer [25]. Over the decades, the region has established a network of public laboratories with the purpose of antivenom production and quality control; this has led to the inception of the Latin American Network of Public Antivenom Manufacturing Laboratories (RELAPA) [58].

The Pan American Health Organisation (PAHO) was established in Latin America and the Caribbean to drive concentrated efforts to deal with public health matters in the Americas and has incorporated SBE into its priorities [84]. Under the guidance of PAHO and its office Centro Panamericano de Fiebre Aftosa (Panaftosa), RELAPA seeks to incorporate governance and technical cooperation, technology transfer, research, and training for the regional improvement of antivenom availability [58]. In Costa Rica, the Caja Costarricense del Seguro Social (CCSS) certifies the quality of the products produced by the Instituto Clodomiro Picado for national use [26]. In Brazil, the Instituto Nacional de Controle de Qualidade em Saúde provides national protocols for the quality control of antivenoms [84].

Most countries in this region rely on centralized acquisition of antivenoms by the Ministries of Health and decentralized or regional distribution to health centers. This is conducted based on the regional needs, assessed through epidemiological data, allowing appropriate stock allocations [26]. The PAHO has developed a software SIGEpi, a tool that is used to track antivenom inventories and improve stocking requirements to drive efficient logistics [26]. The distribution of antivenom throughout the region depends on data-driven stock allocation, digital tracking, and trained medical personnel rather than only cold-chain logistics and procurement [26].

However, there is still a need to strengthen regulatory bodies in countries that rely on imports due to the lack of quality control systems [25]. While there are still some vulnerable areas in Costa Rica, the country has seen an overall decrease in mortality, owing to SBE, due to sustainable public health development, the local manufacture of antivenom, effective management systems, and the training of healthcare personnel [85]. Antivenom is considered an essential medicine; can be prescribed by any physician; and is distributed at the primary, secondary, and tertiary levels. The advantage is especially seen at the primary healthcare level, whereby the time between the SBE incident and the administration of antivenom has shortened significantly [85]. The Clodomiro Picado Institute of Costa Rica is publicly funded and can fulfill national antivenom demands and supply it to Central America, South America, and sub-Saharan Africa [86].

### 4.1. Strengths and Limitations of the Study

This study provided a comprehensive overview of the regulatory landscape and antivenom availability and access globally, with no limitations on the location of publication. The methodology was adopted from the original Arksey and O’Malley framework, with updates from JBI, making it robust. The study selection was reported using the PRISMA statement, adding to the rigor. The PCC criteria were used to develop the screening brief, as this framework provided an excellent approach to the research question.

However, the inclusion criteria were limited to 2009–2023, when SBE remained a public health challenge long before that time, and regulatory policies for antivenom existed beyond it. Due to only articles in English being included, there was an underrepresentation from countries where publications exist in other languages. Additionally, the quality of articles was not assured with any tools. The national regulatory guidelines are not necessarily published in the scientific literature, but also in local manuals and legislation documents. The search strategy of this review did not include these sources of information. While there is published literature on snake antivenom regulations, it may not necessarily meet the current inclusion criteria of this review. Despite the limitations, the search strategy was comprehensive and constructed in collaboration with a health sciences librarian.

### 4.2. Implications of Research and Practice

This study provided limited evidence of regulatory frameworks and policies governing antivenom access and availability in Africa and Asia. Additionally, there is a dearth of information available on standards and policies directed specifically by organizations that regulate medicine production, supply, and quality control. Considering these gaps, there is an urgent need for attention towards SBE and to address the antivenom supply crisis with feasible solutions. More studies are needed that describe and evaluate policies and procedures created by regulatory organizations, especially in SBE endemic areas. Perhaps policymakers and national health authorities should lead with the example of Latin America, especially Brazil and Costa Rica, to facilitate self-sufficient antivenom production, sound distribution systems, and the regional cooperation that has allowed the respective countries to combat mortality resulting from snakebites.

Overall, there is a lack of sufficient evidence of snake antivenom regulations globally. This review may provide a stepping stone for snakebite endemic countries to draw from the gaps and contextualize them to national needs and agendas. It serves as a call to action for local authorities to drive future research and gain collective understanding on regulations guiding antivenom management.

### 4.3. Recommendations for Future Studies

The gaps identified in this study reflect that more research is needed, especially in sub-Saharan Africa and Southeast Asia. More research is recommended to focus on the storage, transport, procurement, and quality control of antivenom. Snakebite endemic countries with chronic shortages of antivenom can use this scoping review to drive focus group interviews with experts, identify unique challenges faced nationally, and compile policy briefs to help find feasible solutions. Future studies should also bring in perspectives of pharmacists and draw attention to prescribing and dispensing practices that affect antivenom access to patients. Additional evidence is required on the feasibility of expired antivenoms and policies governing the use of a valuable resource.

## 5. Conclusions

This study mapped the evidence regarding snake antivenom regulations globally, highlighting the gaps in the literature. With much evidence focused on the impact of manufacturing issues, regulatory oversight, and costs, there was a lack of focus on other aspects pertaining to antivenom access, such as storage, transport, procurement, and quality control. The study also revealed an alarming lack of research from Asia and Africa, which serves as a reflection of the attention SBE receives in these endemic areas. Meanwhile, significant evidence from Latin America showed commendable efforts, through regional cooperation, to reduce the SBE burden. Therefore, further research is recommended. The SBE endemic countries with low publications, especially in Southeast Asia and sub-Saharan Africa, should form focus groups to identify challenges and gaps affecting antivenom access to victims. There is a pressing need for additional investigations into the policies and guidelines present or absent in SBE endemic regions, facilitated by national regulatory authorities.

## Figures and Tables

**Table 1:** The PCC criteria used to develop the search strategy.

		Inclusion	Exclusion
Population	Snake antivenom	Snake antivenoms “**in use**” In use: scheduled products; antivenoms registered currently or historically, such as SAVP polyvalent, monovalent, and global products	Spider antivenom Scorpion antivenom Experimental antivenom Unapproved products
Concept	Accessibility	Regulations (regulatory systems and frameworks) Manufacturing Quality control Procurement Supply chain (availability, transport, procurement, storage) Prescribing and Dispensing	Clinical guidelines related to snakebite treatment
Context	Global	Global	

**Table 2:** Summary of the key findings corresponding to the emerging themes.

Theme	Key Findings
Regulatory frameworks	Role of national health bodies in Latin America Centralized and decentralized distribution systems National programs in India and Thailand Lack of commercial export policy in Africa
Manufacturing and procurement	High-income producers: USA, UK, Japan, Australia, and France Latin America: Brazil, Costa Rica, Argentina, Colombia, and Mexico Asia: India, Thailand, Indonesia, and Philippines Africa: South Africa
Availability and access	Production cessation due to economic constraints Usage of expired antivenom as an alternative resource Impact of cold chain and storage problems on access Poor health infrastructure and geographic inaccessibility High costs

## Data Availability

The raw data supporting the conclusions of this article will be made available by the authors on request.

## Supplementary Materials

The following supporting information can be downloaded at: https://www.mdpi.com/article/10.3390/tropicalmed10080228/s1, Table S1: Comprehensive search strategy for six databases used in the search for publications.

## Abbreviations

The following abbreviations are used in this manuscript:

SBE	Snakebite envenoming
WHO	World Health Organization
PRISMA-ScR	Preferred Reporting Items for Systematic Reviews and Meta-Analysis Extension for Scoping Reviews
CAMs	Complementary and Alternative Medicines
JBI	Joanna Briggs Institute
PCC	Population, concept, context
MeSH	Medical subject headings
CENADI	National Centre for Distribution and Storage of Immunobiologicals
INCQS	National Institute for Quality Control in Health
INVIMA	National Institute for the Surveillance of Medicines and Food
FDA	Food and Drug Administration
GDP	Good distribution practice
GMP	Good manufacturing practice
LMICs	Low- and middle-income countries
