# Correction: Majeed et al. Mapping Evidence on the Regulations Affecting the Accessibility, Availability, and Management of Snake Antivenom Globally: A Scoping Review. *Trop. Med. Infect. Dis.* 2025, *10*, 228

**DOI:** 10.3390/tropicalmed10120350

**Published:** 2025-12-15

**Authors:** Ramsha Majeed, Janette Bester, Kabelo Kgarosi, Morné Strydom

**Affiliations:** 1 Department of Pharmacology, School of Medicine, Faculty of Health Sciences, University of Pretoria, Pretoria 0002, South Africa; morne.strydom@up.ac.za; 2 Department of Physiology, School of Medicine, Faculty of Health Sciences, University of Pretoria, Pretoria 0002, South Africa; janette.bester@up.ac.za; 3 Department of Library Services, Sefako Makgatho Health Sciences, Pretoria 0204, South Africa; kabelo.kgarosi@smu.ac.za

**Error in Table**

In the original publication [1], there was a mistake in Table 2, ‘Summary of the key findings corresponding to the emerging themes’. The original sentence with a mistake is ‘Lack of standardized treatment guidelines and commercial export policy in Africa’, which is in Column 2, Row 2. The corrected sentence is ‘Lack of commercial export policy in Africa’. The revised Table 2 is provided below.

**Text Correction**

There was an error in Section 3.3.1 (Regulatory frameworks), Paragraph 6.

The original sentence with a mistake is ‘The sole antivenom manufacturer in South Africa has no commercial export policy. Additionally, there are no national standardized SBE treatment guidelines [32–34].’ The corrected sentence is ‘The sole antivenom manufacturer in South Africa has no commercial export policy for public scrutiny, according to the included literature [32].’

**References**

Reference number 32 and 34 removed from the paper, so reference 33 in the original version should become 32 in the citation list at the end. Subsequent reference numbers are updated. With this correction, the order of some references has been adjusted accordingly.

The authors state that the scientific conclusions are unaffected. This correction was approved by the Academic Editor. The original publication has also been updated.

## Figures and Tables

**Table 2:** Summary of the key findings corresponding to the emerging themes.

Theme	Key Findings
Regulatory frameworks	Role of national health bodies in Latin America Centralized and decentralized distribution systems National programs in India and Thailand Lack of commercial export policy in Africa
Manufacturing and procurement	High-income producers: USA, UK, Japan, Australia, and France Latin America: Brazil, Costa Rica, Argentina, Colombia, and Mexico Asia: India, Thailand, Indonesia, and Philippines Africa: South Africa
Availability and access	Production cessation due to economic constraints Usage of expired antivenom as an alternative resource Impact of cold chain and storage problems on access Poor health infrastructure and geographic inaccessibility High costs
