# Supplementary material for: Mapping Evidence on the Regulations Affecting the Accessibility, Availability, and Management of Snake Antivenom Globally: A Scoping Review
Source: Trop Med Infect Dis. 2025 Aug 14;10(8):228. doi: 10.3390/tropicalmed10080228 (PMC12389931; doi:10.3390/tropicalmed10080228)
Supplement: Supplementary file 1 [file tropicalmed-10-00228-s001.zip › tropicalmed-3715530-supplementary.pdf]

SUPPLEMENTARY MATERIALS

Supplemental Table S1: Comprehensive search strategy for six databases used in the search for publications.

Database and date	Search	Additional filters	Results
PubMed (07/11/2023)	(("snakes"[MeSH Terms] OR "snakes"[All Fields] OR "snake"[All Fields]) AND ("antivenom*"[All Fields] OR ("antivenins"[MeSH Terms] OR "antivenins"[All Fields] OR "antivenin"[All Fields]) OR "immune sera"[All Fields] OR "anti-snake venom"[All Fields] OR "snakebite therapeutics"[All Fields] OR "serum therapy"[All Fields] OR "antivenom serum"[All Fields]) AND ("drug and narcotic control"[MeSH Terms] OR ("drug"[All Fields] AND "narcotic"[All Fields] AND "control"[All Fields]) OR "drug and narcotic control"[All Fields] OR "regulation*"[All Fields] OR "regulatory framework"[All Fields] OR "regulatory system"[All Fields] OR "manufactur*"[All Fields] OR "quality control"[All Fields] OR "procure*"[All Fields] OR "supply chain"[All Fields] OR ("availabilities"[All Fields] OR "availability"[All Fields] OR "available"[All Fields]) OR ("access"[All Fields] OR "accessed"[All Fields] OR "accesses"[All Fields] OR "accessibilities"[All Fields] OR "accessibility"[All Fields] OR "accessible"[All Fields] OR "accessing"[All Fields]) OR "storage*"[All Fields] OR "prescribe*"[All Fields] OR "dispens*"[All Fields] OR ("pharmacovigilance"[MeSH Terms] OR "pharmacovigilance"[All Fields]) OR "product*"[All Fields] OR "public health challenge"[All Fields] OR "underproduction"[All Fields] OR ("scarcities"[All Fields] OR "scarcity"[All Fields]) OR "pharmacy*"[All Fields] OR "snakebite management"[All Fields] OR "drug policy"[All Fields] OR "distribut*"[All Fields] OR "market demand"[All Fields] OR "pharmaceutical industry"[All Fields] OR "antivenom market"[All Fields] OR "regulatory approval"[All Fields] OR "shelf life"[All Fields] OR ("mortality"[MeSH Terms] OR "mortality"[All Fields] OR "mortalities"[All Fields] OR "mortality"[MeSH Subheading]) OR ("epidemiology"[MeSH Subheading] OR "epidemiology"[All Fields] OR "morbidity"[All Fields] OR "morbidity"[MeSH Terms] OR "morbid"[All Fields] OR "morbidities"[All Fields] OR "morbids"[All Fields]) OR ("epidemiology"[MeSH Subheading] OR "epidemiology"[All Fields] OR "prevalence"[All Fields] OR "prevalence"[MeSH Terms] OR "prevalance"[All Fields] OR "prevalences"[All Fields] OR "prevalence s"[All Fields] OR "prevalent"[All Fields] OR "prevalently"[All Fields] OR "prevalents"[All Fields])) AND ("english"[Language] AND 2009/01/01:2023/12/31[Date - Publication])) AND ((english[Filter]) AND (2009:2023[pdat]))	2009-2023<br>English	1641
SCOPUS<br>(07/11/2023)	(TITLE-ABS-KEY (snakes) AND TITLE-ABS-KEY (antivenom* OR antivenins OR {immune sera} OR {anti-snake venom} OR {snakebite therapeutics} OR {serum therapy} OR {antivenom serum}) AND TITLE-ABS-KEY ({Drug and Narcotic control} OR regulation* OR {regulatory framework} OR {regulatory system} OR manufactur* OR {quality control} OR procure* OR {supply chain} OR availability OR accessibility OR storage* OR prescribe* OR dispens* OR pharmacovigilance OR product* OR {public health challenge} OR underproduction OR scarcity OR pharmacy* OR {snakebite management} OR {drug policy} OR	2009-2023<br>English<br><br>Source type:<br>• Journal<br><br>Document type:<br>• Article<br>Review	1387

	distribut* OR {market demand} OR {pharmaceutical industry} OR {antivenom market} OR {regulatory approval} OR {shelf life} OR mortality OR morbidity OR prevalence)) AND PUBYEAR > 2008 AND PUBYEAR < 2024 AND (LIMIT-TO (SRCTYPE , "j")) AND (LIMIT-TO (DOCTYPE , "ar") OR LIMIT-TO (DOCTYPE , "re")) AND (LIMIT-TO (LANGUAGE , "English"))		
PROQUEST (07/11/2023)	snakes AND (antivenom* OR antivenin* OR "immune sera" OR "anti-snake venom" OR "snakebite therapeutics" OR "serum therapy" OR "antivenom serum") AND ("Drug and Narcotic control" OR regulation* OR "regulatory framework" OR "regulatory system" OR manufactur* OR "quality control" OR procure* OR "supply chain" OR availability OR accessibility OR storage* OR prescribe* OR dispens* OR pharmacovigilance OR product* OR "public health challenge" OR underproduction OR scarcity OR pharmacy* OR "snakebite management" OR "drug policy" OR distribut* OR "market demand" OR "pharmaceutical industry" OR "antivenom market" OR "regulatory approval" OR "shelf life" OR mortality OR morbidity OR prevalence) AND stype.exact("Scholarly Journals") AND at.exact("Feature" OR "Literature Review" OR "Review" OR "Article") AND la.exact("English") AND (at.exact(("Feature" OR "Article" OR "Review" OR "Evidence Based Healthcare" OR "Literature Review") NOT ("Case Study" OR "Report" OR "News" OR "General Information" OR "Undefined" OR "Commentary" OR "Instructional Material/Guideline" OR "Biography" OR "Conference" OR "Correspondence")) AND la.exact("ENG")) AND pd(2009-2023)	2009-2023<br>English<br><br>Source type:<br>• Scholarly journals<br><br>Document type:<br>• Article<br>• Feature<br>• Literature review<br>• Reviews<br><br>Rationale:<br>• Snakebite envenomation was added to WHO list of Neglected Tropical Diseases in 2009.<br>No translators are part of this study.	2203
EBSCO (Africa Wide Information) (07/11/2023)	snakes AND (antivenom* OR antivenin* OR “immune sera” OR “anti-snake venom” OR “snakebite therapeutics” OR “serum therapy” OR “antivenom serum”) AND (“Drug and Narcotic control” OR regulation* OR “regulatory framework” OR “regulatory system” OR manufactur* OR “quality control” OR procure* OR “supply chain” OR availability OR accessibility OR storage* OR prescribe* OR dispens* OR pharmacovigilance OR product* OR “public health challenge” OR underproduction OR scarcity OR pharmacy* OR “snakebite management” OR “drug policy” OR distribut* OR “market demand” OR “pharmaceutical industry” OR “antivenom market” OR “regulatory approval” OR “shelf life” OR mortality OR morbidity OR prevalence)	2009-2023<br>English<br><br>Source type:<br>• Academic journals<br><br>Rationale:<br>• Snakebite envenomation was added to WHO list of Neglected Tropical Diseases in 2009.<br>No translators are part of this study.	86
EBSCO (Academic Search Complete) (7/11/2023)	snakes AND (antivenom* OR antivenin* OR “immune sera” OR “anti-snake venom” OR “snakebite therapeutics” OR “serum therapy” OR “antivenom serum”) AND (“Drug and Narcotic control” OR regulation* OR “regulatory framework” OR “regulatory system” OR manufactur* OR “quality control” OR procure* OR “supply chain” OR availability OR accessibility OR	2009-2023<br>English<br><br>Source type:<br>• Academic journals	1372

	storage* OR prescribe* OR dispens* OR pharmacovigilance OR product* OR “public health challenge” OR underproduction OR scarcity OR pharmacy* OR “snakebite management” OR “drug policy” OR distribut* OR “market demand” OR “pharmaceutical industry” OR “antivenom market” OR “regulatory approval” OR “shelf life” OR mortality OR morbidity OR prevalence)	Rationale:<br>• Snakebite envenomation was added to WHO list of Neglected Tropical Diseases in 2009.<br>No translators are part of this study.	
Web of Science Core Collection (04/10/2023)	snakes (All Fields) and antivenom* OR antivenin* OR “immune sera” OR “anti-snake venom” OR “snakebite therapeutics” OR “serum therapy” OR “antivenom serum” (All Fields) and “Drug and Narcotic control” OR regulation* OR “regulatory framework” OR “regulatory system” OR manufactur* OR “quality control” OR procure* OR “supply chain” OR availability OR accessibility OR storage* OR prescribe* OR dispens* OR pharmacovigilance OR product* OR “public health challenge” OR underproduction OR scarcity OR pharmacy* OR “snakebite management” OR “drug policy” OR distribut* OR “market demand” OR “pharmaceutical industry” OR “antivenom market” OR “regulatory approval” OR “shelf life” OR mortality OR morbidity OR prevalence (All Fields) and Article or Review Article (Document Types) and Science Citation Index Expanded (SCI-EXPANDED) (Web of Science Index) and English (Languages)	2009-2023<br>English<br><br>Science citation index expanded (SCI-EXPANDED)<br><br>Source type:<br>Article<br>Review article<br><br>Rationale:<br>• Snakebite envenomation was added to WHO list of Neglected Tropical Diseases in 2009.<br>• No translators are part of this study.	858
